# Supplementary material for: Identification of system-level features in HIV migration within a host
Source: PLoS One. 2023 Sep 26;18(9):e0291367. doi: 10.1371/journal.pone.0291367 (PMC10521982; doi:10.1371/journal.pone.0291367)
Supplement: S1 File — (PDF) [file pone.0291367.s001.pdf]

Supporting Information: Identification of system-level features in HIV migration within a host

Ravi Goyal[1]
Victor De Gruttola[2]
Sara Gianella[1]
Gemma Caballero[1]
Magali Porrachia[1]
Caroline Ignacio[1]
Brendon Woodworth[1]
Davey M. Smith[1]
Antoine Chaillon[1]

1 Division of Infectious Diseases and Global Public Health, University of California San Diego, La Jolla, CA, US
2 Herbert Wertheim SPH and Human Longevity Science, University of California San Diego, La Jolla, CA, US

Supplementary Table 1: The number of putative breakpoints, their location, and the Akaike Information Criterion derived from a maximum likelihood model fit to each segment.

Last Gift participant	Number of inferred breakpoints	Location (nucleotide positions)	ΔAICc vs the single tree multiple partition*
**LG01**	4 breakpoints	346, 639, 1537, 2337	1412.47
**LG03**	5 breakpoints	383, 581, 995, 1689, 2047	3411.74
**LG04**	3 breakpoints	389, 1050, 1489	1839.02
**LG05**	3 breakpoints	471, 1270, 1761	626.472
**LG06**	3 breakpoints	1205, 1567, 2493	3585.34
**LG08**	8 breakpoints	398, 533, 758, 911, 1205, 1448, 1566, 2119	5839.94
**LG12**	5 breakpoints	367, 573, 819, 1402, 182	1099.12
**LG15**	1 breakpoint	2364	0.00001

*AICc: Akaike Information Criterion derived from a maximum likelihood model fit to each segment. Lower-case ‘c’ indicates that the value has been calculated from the AIC test corrected for small sample sizes. ΔAICc: the improvement in AICc of the best model compared to the single tree model.

Supplementary Table 2: Information on the LG participant number, tissue name, system category, and number of HIV sequences from each tissue sample.

Last Gift participant	Tissue name	System category	Number of HIV sequences
**LG01**	BLOODPLASMA	Blood	7
**LG01**	COLON	Gut	11
**LG01**	ILEUM	Gut	5
**LG01**	LIVER	Others	6
**LG01**	LNPERITRACHEAL	Lymphoid tissues	6
**LG01**	MESENTARYFAT	Others	11
**LG01**	PBMC	Blood	35
**LG01**	PROSTATE	GenitoUrinary Tract	7
**LG01**	SPLEEN	Lymphoid tissues	4
**LG01**	TESTIS	GenitoUrinary Tract	2
**LG03**	BASALGANGLIA	CNS	4
**LG03**	COLONRIGHT	Gut	8
**LG03**	DUODENUM	Gut	7
**LG03**	FRONTALCORTEXMOTOR	CNS	1
**LG03**	HIPPOCAMPUS	CNS	4
**LG03**	ILEUM	Gut	4
**LG03**	LIVER	Others	11
**LG03**	LNAORTIC	Lymphoid tissues	9
**LG03**	LNAXILLARY	Lymphoid tissues	10
**LG03**	LNOTHERS	Lymphoid tissues	6
**LG03**	OCCIPITALCORTEX	CNS	2
**LG03**	PANCREAS	Others	7
**LG03**	PBMC	Blood	21
**LG03**	PERICARDIALADIPOSE	Others	6
**LG03**	PROSTATE	GenitoUrinary Tract	7
**LG03**	RECTUM	Gut	10
**LG03**	SPINALCORD	CNS	9
**LG03**	SPLEEN	Lymphoid tissues	5
**LG03**	TESTIS	GenitoUrinary Tract	3
**LG04**	BASALGANGLIA	CNS	4
**LG04**	BLOODCLOT	Blood	5

**LG04**	BLOODPLASMA	Blood	10
**LG04**	COLONLEFT	Gut	10
**LG04**	COLONRIGHT	Gut	6
**LG04**	DUODENUM	Gut	9
**LG04**	ESOPHAGUS	Gut	7
**LG04**	FRONTALCORTEXMOTOR	CNS	1
**LG04**	HIPPOCAMPUS	CNS	3
**LG04**	ILEUM	Gut	5
**LG04**	JEJUNUM	Gut	15
**LG04**	KIDNEY	GenitoUrinary Tract	7
**LG04**	LIVER	Others	4
**LG04**	LNAXILLARY	Lymphoid tissues	7
**LG04**	LNINGUINAL	Lymphoid tissues	14
**LG04**	LNMESENTARY	Lymphoid tissues	18
**LG04**	OCCIPITALCORTEX	CNS	1
**LG04**	PANCREAS	Others	8
**LG04**	PBMC	Blood	29
**LG04**	PROSTATE	GenitoUrinary Tract	8
**LG04**	RECTUM	Gut	12
**LG04**	SEMINALVESICLE	GenitoUrinary Tract	8
**LG04**	SPINALCORD	CNS	8
**LG04**	SPLEEN	Lymphoid tissues	7
**LG04**	TESTIS	GenitoUrinary Tract	5
**LG05**	BASALGANGLIA	CNS	6
**LG05**	COLON	Gut	1
**LG05**	DUODENUM	Gut	11
**LG05**	FRONTALCORTEXMOTOR	CNS	5
**LG05**	HIPPOCAMPUS	CNS	3
**LG05**	JEJUNUM	Gut	8
**LG05**	LNAORTIC	Lymphoid tissues	10
**LG05**	LNAXILLARY	Lymphoid tissues	1
**LG05**	LNMEDIASTINAL	Lymphoid tissues	3
**LG05**	OCCIPITALCORTEX	CNS	1
**LG05**	PBMC	Blood	5
**LG05**	RECTUM	Gut	2
**LG05**	SPLEEN	Lymphoid tissues	7

**LG06**	BASALGANGLIA	CNS	2
**LG06**	COLONRIGHT	Gut	5
**LG06**	DUODENUM	Gut	6
**LG06**	ESOPHAGUS	Gut	9
**LG06**	FRONTALCORTEXMOTOR	CNS	2
**LG06**	HIPPOCAMPUS	CNS	2
**LG06**	ILEUM	Gut	8
**LG06**	JEJUNUM	Gut	7
**LG06**	LIVER	Others	10
**LG06**	LNAORTIC	Lymphoid tissues	9
**LG06**	LNAXILLARY	Lymphoid tissues	13
**LG06**	LNMEDIASTINAL	Lymphoid tissues	5
**LG06**	PANCREAS	Others	3
**LG06**	PBMC	Blood	33
**LG06**	PROSTATE	GenitoUrinary Tract	3
**LG06**	RECTUM	Gut	5
**LG06**	SEMINALVESICLE	GenitoUrinary Tract	6
**LG06**	SPINALCORD	CNS	3
**LG06**	SPLEEN	Lymphoid tissues	7
**LG06**	TESTIS	GenitoUrinary Tract	6
**LG08**	BASALGANGLIA	CNS	1
**LG08**	COLONRIGHT	Gut	1
**LG08**	DUODENUM	Gut	4
**LG08**	EPIDIDYMUS	GenitoUrinary Tract	6
**LG08**	ESOPHAGUS	Gut	6
**LG08**	FRONTALCORTEXMOTOR	CNS	1
**LG08**	ILEUM	Gut	6
**LG08**	JEJUNUM	Gut	7
**LG08**	LIVER	Others	6
**LG08**	LNAORTIC	Lymphoid tissues	6
**LG08**	LNHILAR	Lymphoid tissues	5
**LG08**	LNHILAR	Lymphoid tissues	5
**LG08**	OCCIPITALCORTEX	CNS	2
**LG08**	PANCREAS	Others	6
**LG08**	PBMC	Blood	14

**LG08**	PROSTATE	GenitoUrinary Tract	6
**LG08**	RECTUM	Gut	9
**LG08**	SEMINALVESICLE	GenitoUrinary Tract	9
**LG08**	SPINALCORD	CNS	3
**LG08**	SPLEEN	Lymphoid tissues	6
**LG08**	TESTIS	GenitoUrinary Tract	7
**LG12**	COLONRIGHT	Gut	5
**LG12**	DUODENUM	Gut	6
**LG12**	ILEUM	Gut	5
**LG12**	JEJUNUM	Gut	7
**LG12**	PBMC	Blood	24
**LG12**	RECTUM	Gut	11
**LG12**	SPLEEN	Lymphoid tissues	9
**LG15**	BASALGANGLIA	CNS	10
**LG15**	COLONRIGHT	Gut	7
**LG15**	DUODENUM	Gut	11
**LG15**	FRONTALCORTEXMOTOR	CNS	5
**LG15**	HIPPOCAMPUS	CNS	9
**LG15**	ILEUM	Gut	9
**LG15**	JEJUNUM	Gut	10
**LG15**	OCCIPITALCORTEX	CNS	2
**LG15**	PBMC	Blood	5
**LG15**	RECTUM	Gut	8
**LG15**	SPINALCORD	CNS	3
