# Supplementary material for: Identification of system-level features in HIV migration within a host
Source: PLoS One. 2023 Sep 26;18(9):e0291367. doi: 10.1371/journal.pone.0291367 (PMC10521982; doi:10.1371/journal.pone.0291367)
Supplement: Attachment — Submitted filename: ViralMigrationNetwork_responses_07_05_2023.pdf [file pone.0291367.s002.pdf]

Dear Dr. Nafees Ahemad,

We are grateful to the reviewers for the helpful comments about our paper "Identification of system-level features in HIV migration within a host"' (PONE-D-22-24111). Below we address the concerns raised by the reviewers. The reviewers' comments are cited in italics.

Reviewer #1:

1. *Goyal and colleagues utilize unique and diverse tissue samples from the Last Gift cohort to assess HIV DNA sequencing data to study the "migration" of HIV sequences across and between tissues. This is an interesting methodological and data analysis approach to questions that are difficult to ask, especially as access to tissues is limited. The approaches have several technical limitations, which may currently be unavoidable, that should be acknowledged and discussed.*

Response:

In the revised manuscript, we included additional discussion on limitations of the data and methods. Specifically, we added limitations on:

- Our use of envelope sequences compared to full-length genome (see our response to your comment below).
- The potential impact of viral recombination (see our response to Reviewer 2).
- The generalizability of our findings due to heterogeneity in the Last Gift cohort participants (see our response to Reviewer 2).

The original submission and revised submission discuss the following limitations:

- Convergence issues of the statistical network model (ERGMs)—this issue has been well-studies in previous literature.
- The limited number of Last Gift cohort participants.
- The fact that further research is necessary to identify mechanisms driving viral migration.

2. *As has been recently discussed (White JA et al. PLoS Pathog. 2022. PMID: 36074794) near-full-length PCR techniques may introduce bias and over-represent some sequences. How could such bias influence the representation of sequences and their apparent "flow" or change in proportion over time? Further most sequences are defective, but may proliferate as their host cells proliferate, Both myeloid and lymphoid cells may carry HIV DNA, and some cells may migrate within tissue to various body compartments, without true migration of viral particles due to spreading infection. These biological aspects should be discussed in the methodological description of the analysis and its interpretation.*

Response:

We thank the reviewer for raising this concern. This is indeed an important aspect to consider for near full-length genome (NFLG) sequencing methods. As nicely described by White et al (2022);[1] NFLG may miss a large proportion of intact proviruses due to amplification failure at the initial outer PCR step. In our study, we did not sequence NFLG but the FL envelopes (gp160) using single genome dilution techniques. We also include a filtering step to identify of defective or hypermutant sequences.[2] While NFLG sequencing methods rely on a long-distance outer PCR capturing most (~9 kb) of the genome, our analyses include FL envelopes and are therefore less likely to be impacted by amplification failure of long fragment (9kb). While we are not implementing NFLG sequencing techniques, we agree with White et al (2022)that IPDA methods or equivalent are important techniques to quantify and distinguish intact from defective proviruses without the need for long-distance PCR.[1]

We included these points in the Discussion Section; the additional text is below in red:

> *"Our analyses have several other scientific limitations. The analyses are conducted on 8 participants, some of whom had a limited number of tissues. Also, there is heterogeneity in the participants, in particular, their terminal disease and ART usage; this heterogeneity may impact the generalizability of our findings. In addition, the construction of the VMNs is based on HIV full-length envelopes sequences (gp160) using single genome dilution techniques. We also include a filtering step to identify of defective or hypermutant sequences.[2] While using HIV envelopes contain less information than full-length genome, they are less likely to be impacted by amplification failure of long fragment. Furthermore, full-length genome may miss a large proportion of intact proviruses due to amplification failure.[1]"*

Reviewer #2:

1. *The paper states that data and software will be available from the authors upon request, but the proper way to make the data available is to create GenBank entries for the HIV sequences and list the accession numbers in the publication. It is also very nice of multiple sequence alignments, or other useful data formats are stored at TreeBase, or the data DRYAD or similar online repositories.*

Response:

We have uploaded the sequences on dryad and will be made publicly available at the time of publication.

2. *Intra-patient viral recombination is not mentioned in the paper. It is difficult to analyze intra-subtype recombination in HIV-1, and even more difficult to analyze intra-patient recombination. However, tools such as the Highlighter too at the LANL HIV Database (https://www.hiv.lanl.gov/content/sequence/HIGHLIGHT/highlighter_top.html) and*

GARD (http://www.datamonkey.org/GARD/) can be useful in identifying whether or not recombination is likely to be influencing phylogenetic analyses.

Response:

We agree with the reviewer that recombination is an important aspect to consider as it shapes HIV evolution. If compartmentalization reflects spatial segregation of the virus population, viral recombination is a result of mixing of the population. Hence, if different point mutations may arise in different tissues, viral migration may brings these variants together and lead eventually to recombination and intermixed viral population. We acknowledge that both migration and recombination shoud be investigated when studying HIV-1 dynamics within host. Our study does not attempt to evaluate this combined effect and it would require further investigation.[3] Other factors such as different local immune pressure, antiretroviral therapies, etc would also need to be considered to comprehensitively characterize factors influencing viral dynamics and evolution within host.

As suggested by the reviewer, we evaluated the potential impact of intra-host recombination. First, we used GARD to identify potential recombination breakpoints.[4] See the table below for an overview of the number and position (relative to HXB2 *env*) of inferred breakpoint identified.

ID	Number of inferred breakpoints	Location (nucleotide positions)	ΔAICc vs the single tree multiple partition*
LG01	4 breakpoints	346, 639, 1537, 2337	1412.47
LG03	5 breakpoints	383, 581, 995, 1689, 2047	3411.74
LG04	3 breakpoints	389, 1050, 1489	1839.02
LG05	3 breakpoints	471, 1270, 1761	626.472
LG06	3 breakpoints	1205, 1567, 2493	3585.34
LG08	8 breakpoints	398, 533, 758, 911, 1205, 1448, 1566, 2119	5839.94
LG12	5 breakpoints	367, 573, 819, 1402, 182	1099.12
LG15	1 breakpoint	2364	0.00001

*AICc: Akaike Information Criterion derived from a maximum likelihood model fit to each segment. Lower-case ‘c’ indicates that the value has been calculated from the AIC test corrected for small sample sizes. ΔAICc:the improvement in AICc of the best model compared to the single tree model;

Next, to evaluate the potential impact of recombination on the migration analyses, we reran our network models (i.e., exponential random graph models [ERGMs]) using partitioned datasets according to the inferred breakpoint(s). These new models exhibited convergence issues. Therefore, we can not provide conclusive assessment of the impact of recombination on features of the viral migration network. In the original submission, we provided additional details regarding convergence issues with ERGMs, which are well-studied in network literature. In addition, we provided information on an alternative network model that does not exhibit such issues, but requires additional methodological development to address missing network

data—which is necessary for our context of missing tissues; such methodological development is currently underway.

We included these points in the Discussion Section; the additional sentences are below in red:

> *"Evolution of HIV-1 in a host is shaped by many evolutionary forces, including recombination. If compartmentalization reflects spatial segregation of the virus population, viral recombination is a result of population mixing. Hence, if different point mutations may arise in different tissues, viral migration may bring these variants together and lead eventually to recombination and intermixed viral population. We acknowledge that both migration and recombination should be investigated when studying HIV-1 dynamics within host. While our study does not attempt to evaluate this combined effect and it would require further investigation,[3] we investigate the potential impact of intra-host recombination. To do so, we first use GARD to identify potential recombination breakpoints;[4] see Supplementary Table 1 in Supplementary Materials for an overview of the number of putative breakpoint identified. Next, we run our network models (i.e., ERGMs) using the partitioned dataset according to the inferred breakpoint(s). However, these new models exhibit convergence issues; therefore, we cannot provide conclusive assessment of the impact of recombination on features of the viral migration network. Below we provide additional details regarding convergence issues with ERGMs and a alternative network model that does not exhibit such issues, but requires additional methodological development. Furthermore, other factors, such as local immune pressure and antiretroviral therapies, would also need to be considered to comprehensively characterize factors influencing viral dynamics and evolution within host."*

3. *The Figure 1 legend does not mention it, but I assume the overall size or diameter of each patient graph is proportional to virus diversity in that patient. So for example LG12 fig1G had less diverse virus than LG01 fig1A.*

Response:

We have clarified that the size of the tissues in Figure 1 refer to the number of sequences in each tissue.

We included this information in the Result Section and Caption of Figure 1; the additional text is below in red:

> *Results Section: "The VMNs based on DTA applied to the LG cohort are shown in Figure 1. The nodes and edges represent tissues and migration events among the tissues, respectively. The node color indicates the biological system to which the tissue belongs (for example, central nervous system or gut), the node size is proportion to the number of sequences within the sample, and the edge width denotes the number of migrations between the two tissues connected."*

Figure 1 Caption: *"Visual representation of the VMNs for each of the Last Gift participants. The nodes and edges represent individual tissues and migration events among the tissues, respectively. The node color indicates the biological system to which the tissue belongs, the node size is proportion to the number of sequences within the sample, and the edge width denotes the number of migrations between the two tissues."*

4. *There are many sentences which don't make sense to me, and I wonder if it is because words are missing? For example on page 15 "While phylodynamic modeling has greatly enhanced these e orts, our analysis provides additional insights through analysis of migration as a network | rather than as pairwise events tissue." Maybe was supposed to end with "pairwise events between two tissues."?*

Response:

We reviewed the entire paper and clarified several sentences. In particular, we revised the example you provided (your suggested revision is what we intended); the revision is provided below (red indicates our changes):

"While phylodynamic modeling has greatly enhanced these efforts, our analysis provides additional insights through analysis of migration as a network—rather than as pairwise events between two tissues."

5. *I think the paper could benefit from a better description of how this type of study can help with a cure. The paper says "Insights gained using network science techniques in analysis of VMNs have therapeutic implications, in that they may aid in the identification of common features in viral migration, and, by facilitating the targeting of specific processes, potentially increase efficacy of HIV cure." Three of the 8 patients were not on ART at the time of death, and might have therefor had higher viral loads than the others.*

Response:

We elaborated on how our study can help with a cure; the additional/modified text is below in red:

"The primary goal of our results and investigation is to understand the potential (and necessity) of analyzing viral migration using network science techniques. While phylodynamic modeling has greatly enhanced these efforts, our analysis provides additional insights through analysis of migration as a network—rather than as pairwise events between two tissues. Insights gained using network science techniques in analysis of VMNs have therapeutic implications, in that they may aid in the identification of common features in viral migration in people with HIV (or a subpopulation, such as those who interrupt ART). An understanding of these features may elucidate potential processes to target the source of viral reseeding. For example, our findings suggest a hierarchical structure for viral migration among the tissues. Treatments targeting tissues

upstream may be more efficient in preventing viral rebound compared to treatments focused on tissues further down in the viral migration structure. Therefore, this research may serve as initial insight into developing more efficient treatments to provide viral migrating and reseeding of tissues."

6. *The paper has quite a bit of discussion of the computational analyses, but no information is provided about the data acquisition.*

    a. *How were the tissues sampled to reduce or eliminate the potential for sampling blood cells rather than tissue cells in each tissue?*

Response:
We agree that blood contamination is a concern when collecting tissue samples during autopsy. No tissues were included that gross blood contamination during autopsy, but we cannot completely exclude the possibility of some blood contamination. This is likely to be a small impact on our analysis given the small size of capillaries compared to overall tissue mass. Our evaluation of methods to flush blood from organs during the autopsy has shown only limited ability to remove blood cells given settling of blood after death (livor mortis). While we cannot completely rule out such contamination, our sequence analyses showed viral compartmentalization for all participants, which suggest that the putative blood contamination did not significantly impact our analyses.

We included these points in the Discussion Section; the additional text is below in red:

"Our findings also can be impacted by blood T cell contamination of tissue samples obtained during autopsy. Previous sequence analyses on the samples showed viral compartmentalization for all participants, which suggests that possible blood contamination would not negate our findings; see Chaillon et al (2020) for additional details regarding contamination."

    b. *Are the sequences likely to be from viral RNA or from proviral DNA integrated into the host genome? Was the complete envelope gp160 region sequenced?*

Response:

All but blood plasma samples are proviral DNA sequences. The genomic DNA was extracted from 5 million PBMCs and snap-frozen tissues using QIAamp DNA Mini Kit (Qiagen cat#51306) per manufacturer's protocol. After extraction, precipitation was performed to concentrate DNA. Concentrations of DNA were determined using NanoDrop One (ThermoScientific). We performed single genome dilution and sequencing of full length envelope (gp160). Additional details are provided in Chaillon et al (2022).

We included a summary of these points in the Method Section; the additional text is below in red:

"Table 3 presents the demographic characteristics of the participants and summary statistics of their VMN, including the number of observed and missing tissues and the number of directed edges (i.e., the presence of a migration event inferred from Bayesian models). Supplementary Table 2 provides information on the LG participant number, tissue name, system category, and number of HIV sequences from each tissue sample. All HIV sequences—except for blood plasma samples—are proviral DNA sequences. The genomic DNA is extracted, and precipitation is performed to concentrate DNA. Concentrations of DNA are determined using NanoDrop One (ThermoScientific). We perform single genome dilution and sequencing of full-length envelope (gp160). See the supplementary materials in Chaillon et al (2020) for additional information on collection and processing of data from the LG Cohort."

c. *What were the 33 tissues for each patient? Table 3 shows 7 tissues sampled and 26 missing for patient LG12 and Gig 1G shows 7 nodes with 5 of them being from gut. Most patients seem to have just one or two blood tissues sampled. In many places, the paper says "number of HIV sequences" but nowhere is it mentioned whether there ere hundreds of sequences from each tissue sample, or dozens, or thousands.*

Response:

We include a supplementary table that contains the data on the number of HIV sequences from each tissue sample as well as the LG participant number, tissue name, and system associated with the sample.

We included a reference to the supplementary table in the Methods Section; the additional text is below in red:

"Supplementary Table 2 provides information on the LG participant number, tissue name, system category, and number of HIV sequences from each tissue sample."

The caption for the Supplementary Table 1 is the following:

"Supplementary Table 2: Information on the LG participant number, tissue name, system category, and number of HIV sequences from each tissue sample."

References

[1] Jennifer A White, Joshua T Kufera, Niklas Bachmann, Weiwei Dai, Francesco R Simonetti, Ciara Armstrong, Jun Lai, Subul Beg, Janet D Siliciano, and Robert F Siliciano. Measuring the latent reservoir for hiv-1: Quantification bias in near full-length genome sequencing methods. PLoS pathogens, 18(9):e1010845, 2022.

[2] Antoine Chaillon, Sara Gianella, Simon Dellicour, Stephen A Rawlings, Timothy E Schlub, Michelli Faria De Oliveira, Caroline Ignacio, Magali Porrachia, Bram Vrancken, Davey M Smith, et al. Hiv persists throughout deep tissues with repopulation from multiple anatomical sources. The Journal of clinical investigation, 130(4):1699–1712, 2020.

[3] Sumantra Sarkar, Ethan Romero-Severson, and Thomas Leitner. Migration coupled with recombination explains disparate hiv-1 anatomical compartmentalization signals. bioRxiv, pages 2023–04, 2023.

[4] Sergei L Kosakovsky Pond, David Posada, Michael B Gravenor, Christopher H Woelk, and Simon DW Frost. Automated phylogenetic detection of recombination using a genetic algorithm. Molecular biology and evolution, 23(10):1891–1901, 2006.
